# Injury Profiles Associated with Artisanal and Small-Scale Gold Mining in Tarkwa, Ghana

**DOI:** 10.3390/ijerph120707922

**Published:** 2015-07-10

**Authors:** Benedict N. L. Calys-Tagoe, Lauretta Ovadje, Edith Clarke, Niladri Basu, Thomas Robins

**Affiliations:** 1 Department of Community Health, School of Public Health, University of Ghana, Legon Boundary, Accra, Ghana; E-Mail: calys75@hotmail.com; 2 Department of Environmental Health Sciences, School Of Public Health, University of Michigan, Ann Arbor, MI 48109, USA; E-Mails: lovadje@umich.edu (L.O.); trobins@umich.edu (T.R.); 3 Ghana Health Service, Accra, Ghana; E-Mail: essieclarke@yahoo.com; 4 Faculty of Agricultural and Environmental Sciences, McGill University, CINE Building, Macdonald Campus of McGill University, 21111 Lakeshore Rd., St. Anne de Bellevue, QC H9X 3V9, Canada

**Keywords:** small-scale gold mining, ASGM, injuries, accidents, Ghana, Tarkwa mining district, occupational health, mining

## Abstract

Artisanal and small-scale gold mining (ASGM) is inherently risky, but little is known about mining-associated hazards and injuries despite the tremendous growth worldwide of ASGM and the benefits it offers. The current study aimed to characterize the physical injuries associated with ASGM in Ghana to guide policy formulation. A cross-sectional survey was carried out in the Tarkwa mining district of the Western Region of Ghana in 2014. A total of 404 small-scale miners were recruited and interviewed regarding their occupational injury experiences over the preceding 10 years using a paper-based structured questionnaire. Nearly one-quarter (23.5%) of the miners interviewed reported getting injured over the previous 10 years, and the overall injury rate was calculated to be 5.39 per 100 person years. The rate was significantly higher for women (11.93 per 100 person years) and those with little mining experience (e.g., 25.31 per 100 person years for those with less than one year of work experience). The most injury-prone mining activities were excavation (58.7%) and crushing (23.1%), and over 70% of the injuries were reported to be due to miners being hit by an object. The majority of the injuries (57%) were lacerations, and nearly 70% of the injuries were to the upper or lower limbs. Approximately one-third (34.7%) of the injuries resulted in miners missing more than two weeks of work. One-quarter of the injured workers believed that abnormal work pressure played a role in their injuries, and nearly two-fifths believed that their injuries could have been prevented, with many citing personal protective equipment as a solution. About one-quarter of the employees reported that their employers never seemed to be interested in the welfare or safety of their employees. These findings greatly advance our understanding of occupational hazards and injuries amongst ASGM workers and help identify several intervention points.

## 1. Introduction

The mining industry is a vital economic sector for many countries, particularly those in the low- and middle-income economic brackets. The global demand for export products, as well as investment capital coupled with the geological endowment of the West African sub-region has led to the establishment of several multi-national gold mining companies in the area [1]. Ghana, a country in the sub-region, has become a preferred destination for mineral sector investment, with the legal mining industry contributing over 49% of the country’s gross foreign exchange earnings in 2010 [2]. 

It has been long known that mining is one of the most hazardous work environments [3,4]. A typical mining operation involves exposures of workers to a range of hazards, including extreme temperatures, injuries from machineries and other objects and falls [5]. Due to their severity and frequency, injuries and fatalities within the mining sector are amongst the most expensive in terms of the average cost per worker [6]. 

Informal mining poses even more hazards than what may be found in a highly-organized and/or regulated large-scale operation. For example, the International Labour Organization [5] estimates that non-fatal accidents are 6–7-times more common in informal mining operations when compared to large-scale operations. In Ghana, small-scale (gold) mining is defined as “…mining (gold) by any method not involving substantial expenditure by an individual or group of persons not exceeding nine in number or by a co-operative society made up of ten or more persons” [7]. This definition encompasses what is termed “artisanal”, in other words operations that use rudimentary implements, as well as mining that involves more sophisticated activities, but operating at a relatively low level of production and requiring limited capital investment. This is the definition adopted for the purposes of the current study. 

Despite the fact that each individual small-scale mining operation is by definition limited in size, owing to the very large numbers and high concentration of such operations, their combined economic and social impact is significant in many developing countries, including Ghana. For example, a report from 1999 estimated that within Sub-Saharan Africa, small-scale mining produces gold and gemstones worth about $1 billion annually [5], which is likely a great under-estimation given the tremendous recent growth of these industries across the continent. The small-scale gold mining sector provides employment for a large proportion of the unemployed in the areas where it exists and currently employs between 500,000 and one million people in Ghana, predominantly in rural areas [8]. Thus, small-scale gold mining is of tremendous economic importance in Ghana and many other developing countries, especially in places where alternative livelihoods are limited [9].

These economic benefits notwithstanding, small-scale gold mining is faced with major challenges, particularly in the area of occupational health and safety [10]. In the vast majority of settings where small-scale mining occurs, it is characterized by a lack of long-term planning and use of rudimentary techniques [11]. People in the sector operate under dangerous, labor-intensive, disorganized and insecure conditions. These conditions may be expected to increase the risk of occupational injury, especially for those mining without any form of monitoring. In addition to the potentially hazardous environment in which mine workers operate, they appear to be at increased risk of conditions and diseases, such as alcoholism and sexually-transmitted infections (STIs), not directly related to occupational risk factors [9,12].

A number of studies have characterized mining-associated hazards and injuries, though most have been situated in developed countries and have focused on large-scale mining operations. There is very limited research addressing occupational hazards and injuries with respect to small-scale mining operations in low- and middle-income countries. In Ghana, for example, a survey of 46 miners in the Kumasi region revealed that most were concerned about collapses and falls [13]; a survey of workers in the Wassa West District found that approximately 47% reported occupational injuries over the past 10 years [14]; and a study in the Upper East Region found that of 120 miners, 70% indicated that they never use any form of personal protective equipment [15]. These descriptive studies provide some background, though as the small-scale gold mining sector continues to grow worldwide, there is a pressing need for hazard and injury factors to be quantitatively and robustly studied so that findings may guide policy formulation with the aim of protecting the health and safety of this vulnerable group of workers. Furthermore, within the UNEP Minamata Convention on Mercury, there exists a special mention (Annex C) on implementing public health strategies to prevent hazardous exposures amongst vulnerable populations with artisanal and small-scale gold mining communities. With these in mind, the current study aimed to characterize the physical injuries associated with small-scale gold mining in Ghana. Specifically, a cross-sectional study was conducted in the Tarkwa region to characterize the socio-demographics of small-scale miners, work activities and duration, work-related injuries (including causes, types, localization, severity and rates), training activities and employer attitudes towards safety.

## 2. Experimental Section 

### 2.1. Study Location

The study was undertaken in the Tarkwa-Nsuaem municipality and the Prestea-Huni Valley district, both within the Tarkwa mining district of the Western Region of Ghana. Small-scale gold mining activities have been ongoing in this area for several decades [16], and there is a well-established community of small-scale gold miners [17]. 

### 2.2. Study Design and Population

A cross-sectional survey was carried out between March and April of 2014. Mining companies were identified within the Tarkwa-Nsuaem municipality and Prestea-Huni Valley district. A total of 404 small-scale gold miners were recruited from 9 small-scale gold mining sites, 5 of which were licensed and the others unlicensed. All mineworkers present at the selected sites during the study period were invited to participate in this study. Ethical approval for the study was obtained from the Ethics Review Committee of the Ghana Health Service (GHS) (ID No. GHS-ERC: 17/03/14) and the Institutional Review Board (IRB) of the University of Michigan (ID No. HUM00085165). 

The licensed mines were selected by simple random ballots from a list of functional licensed mines within the study area. The unlicensed mines were identified using the concept of creating “contact zones”. This concept is often used in the context of highly asymmetrical relations of domination and subordination, especially among people with unusual power relations [18]. This approach was necessary because of the general perception about small-scale mining in Ghana. Governmental and public discourse, as well as the Ghanaian media has often portrayed them in a negative manner [10]. The “contact zones” therefore created a conducive environment for the establishment and maintenance of rapport and trust between the research team and the leaders of the miners, which involve elaborate explanations of the research and its benefits. The leaders in turn assisted the research team in the recruitment of subjects for the study, since they know and have the trust of the various mine workers. 

### 2.3. Data Collection

A structured, paper-based, interviewer-administered questionnaire was used to obtain needed information from the miners. Information obtained included demographic characteristics (age, sex and educational background), mining history (duration of working in mining, previous and current place of work, registration status of work places, mining activities engaged in) and small-scale gold mining workplace injury history for the 10 years preceding the date of interview (place of injury, type of injury, body part affected during injury, cause of injury, severity of injury). A 10-year timeframe was chosen to balance a desire to obtain sufficient data on injuries, so as to ensure adequate statistical power, *versus* the diminishing accuracy of recalling events from years ago. The interviews were conducted by indigenes of the study area who were well versed in both English and Twi (the local dialect), had completed a minimum of secondary education and were trained specifically for this study. Each completed questionnaire was cross-checked by the Principal Investigator on the field to ensure completeness. 

### 2.4. Data Management and Analysis

Data were captured electronically using SPSS Version 22 for Windows and double-keyed to ensure consistency. Data analysis was done using SPSS Version 22 and Microsoft Excel 2013. Descriptive statistics (frequencies, proportions, rates, means and standard deviations) were carried out. Associations between study variables were assessed using Pearson’s chi-square, ANOVAs and correlations as required.

## 3. Results 

### 3.1. Socio-Demographics

A total of 404 small-scale gold miners operating in the Tarkwa-Nsuaem municipality and the Prestea-Huni Valley districts, both within the Tarkwa mining district, were interviewed. Over 90% of the participating miners were male, though 32 females were included, and here, we report on them separately in certain cases. Of all of the miners, nearly 75% were less than 40 years old with a mean age of 33.8 ± 10.6 years and a median of 32 years (range: 17–72 years) (Table 1). Self-reported tobacco use was minimal among these miners, with only 3.5% who indicated that they currently smoked tobacco and almost 90% who had never smoked tobacco. Among those who smoked, the mean number smoked per day was four cigarettes. Among the 44% who reported drinking alcohol within the past 12 months, 60% reported consuming an average of one drink per day, with 25%, 7.9% and 4.3% consuming 2, 3 and 4 drinks daily, respectively. No one reported consuming more than four drinks in a day. Note, standard drink units were adopted from the U.S. National Institute on Alcohol Abuse and Alcoholism. There were no associations between smoking or drinking alcohol with the outcomes described below.

**Table 1:** Socio-demographic characteristics of the study participants.

Variables	% of Respondents
**Age groups (years); N = 404**	
<19	3.2
20–29	40.1
30–39	30.4
40–49	17.3
50–59	6.9
60–69	1.7
>70	0.2
**Sex; N = 404**	
Male	92.1
Female	7.9
**Educational attainment; N = 404**	
% completed senior high school	28.5
**Marital status; N = 404**	
Living with partner	65.8
Living without partner	34.2
**Tobacco use; N = 318**	
Never smoked	89.9
Ever smoked	10.1
Currently smokes	3.5
**Alcohol consumption in the last 12 months; N = 318**	
Yes	43.4
No	56.6

In the study, 32 females were interviewed. Their ages ranged from 21–55 with a median value of 38 years. Like the males, most females (23/32) self-identified as Akan. Only three of the women reported to be single, with the rest being married or cohabiting (22), widowed (five) or separated (two). This was significantly different from the males in which 93 (of 372) were single. In terms of education, more than 50% of the females (18/32) reported to have never attended school, and this was again significantly different from the males, in which only 24 (6.5%) reported to have never attended school. Of the females who attended school, only one (3.1%) completed secondary school *versus* 28.9% of the males. None of the women reported to currently smoke, and five of them reported drinking alcohol.

### 3.2. Work Duration and Activities

Reported time having worked in the small-scale mining industry by the participants ranged from one month–30 years, with a mean work duration of 6.4 ± 6.6 years. The median number of years worked was four, with the 25th and 75th percentiles being 1.1 and 10 years, respectively. Twenty two percent (22%) reported working in the industry for more than 10 years. The majority (61.9%) had worked at only one site throughout their mining career. In terms of reported time having worked in their current small-scale mining workplace, the responses ranged from one month–24 years with a mean value of 4.2 ± 5.1 years and a median value of two years. Over the last 10 years (which is the time period for which participants were queried about experiencing injuries), the 404 study participants worked a total of 2245 person years in the small-scale gold mining sector. The work duration for the females interviewed ranged from one month–14 years with a mean and median value of 4.5 and three years, respectively. 

**Figure 1:**
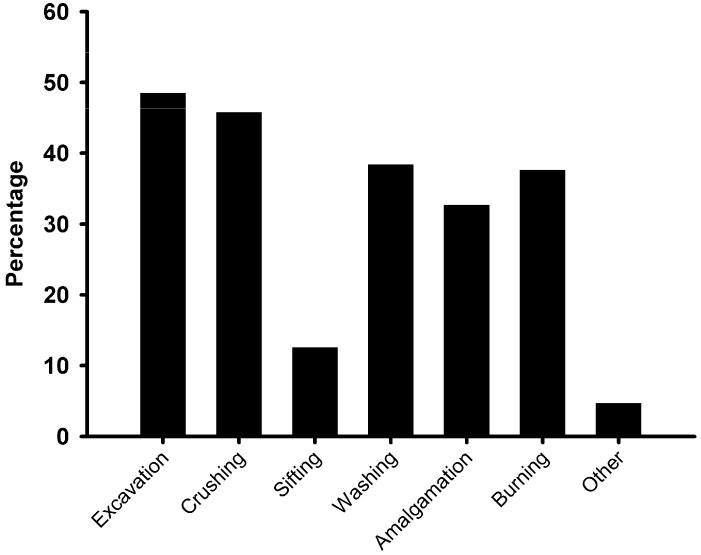
Self-reported routine activities of artisanal and small-scale gold miners from the Tarkwa (Ghana) study sites. Note, cumulative values exceed 100%, since many miners are regularly involved in more than one activity.

All participants were involved in one or more of the six main small-scale mining activities, namely excavation, crushing and grinding, sifting and shanking, washing and sluicing, amalgamation and burning (Figure 1). Over 50% of them were involved in more than one of these activities. Nearly 50% were involved with excavation and/or crushing, and approximately one-third were involved with washing, amalgamation and burning. Less than five percent were involved in other non-core mining activities, such as accounting, security, water pump operation, welding and supervision. Activities varied according to the age of the respondent and the number of years for which they had mined (Table 2). Furthermore, of the 32 females interviewed, three responded “yes” to excavating, nine responded “yes” to crushing ore and 20 responded “yes” to carrying loads (note, of these, two women responded “yes” to doing both excavating and carrying loads). Two of the women responded “yes” to washing, amalgamating and burning. No other job categories were recorded for the women.

**Table 2:** Percentage of miners participating in the key artisanal and small-scale gold mining activities in the Tarkwa (Ghana) study sites according to age and duration of mining. Note, rows do not add up to 100%, since many miners are regularly involved in more than one activity.

		Excavation	Crushing	Sifting	Washing	Amalgamation	Burning
**Age Categories (Age Range)**	10–19 (n = 13)	15.4	38.5	0	30.8	30.8	30.8
	20–29 (n = 162)	49.4	48.8	16.0	39.5	30.9	35.8
	30–39 (n = 123)	53.7	45.5	12.2	40.7	37.4	39.0
	40–49 (n = 70)	47.1	42.9	11.4	35.7	30.0	42.9
	50–59 (n = 28)	42.9	42.9	3.6	28.6	28.6	32.1
	60–69 (n = 7)	28.6	42.9	14.3	42.9	28.6	42.9
	70–79 (n = 1)	0	0	0	100	100	0
**Mining Experience (Years)**	≤5 (n = 246)	41.9	43.5	8.9	29.3	21.1	25.2
	6–10 (n = 71)	49.3	49.3	16.9	47.9	45.1	50.7
	>10 (n = 87)	66.7	49.4	19.5	56.3	55.2	62.1

### 3.3. Injury Experience, Causes and Severity

Within the last 10 years, 95 of the 404 individuals interviewed experienced an injury severe enough to make them miss days of work or hamper their ability to work effectively when they showed up to work. The calculated incidence proportion of injury was 23.5%. The majority of them (75/95) experienced a single injury episode during the period. The number of miners experiencing 2, 3, 4 and 5 injuries in last 10 years were 13, 5, 0 and 1, respectively, for a total of 121 injury episodes. With a total of 121 injuries and 2,245 person years of mining work within the last 10 years, the overall injury rate was calculated at 5.39 per 100 person years. The injury rate for women was calculated to be 11.93 injuries per 100 person years (14 injury episodes over 117 person years), which was significantly higher than that for the men (2.73 per 100 person years). 

**Figure 2:**
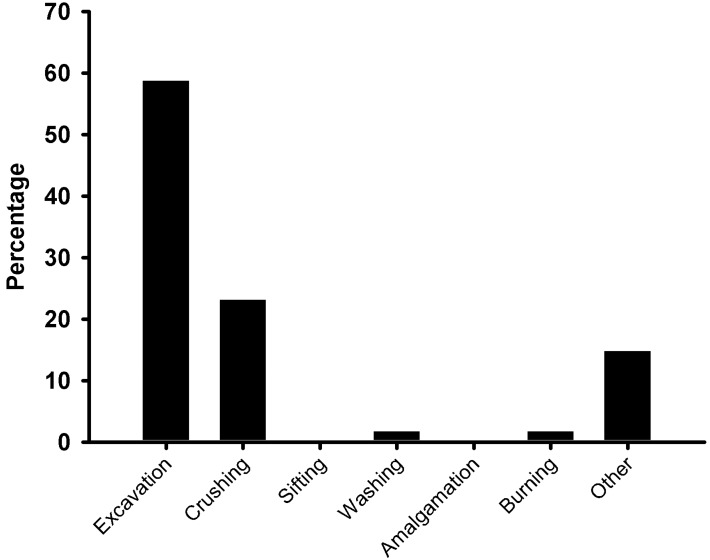
Self-reported activities of artisanal and small-scale gold miners from the Tarkwa (Ghana) study sites at the time of injury.

The occurrence of injury was significantly associated with the activity of the miner at the time of injury (*p* < 0.001). Excavation (58.7%) and crushing (23.1%) were the most injury-prone activities, while no injuries were recorded during the sifting or amalgamation process (Figure 2). The occurrence of injury was found to be significantly associated with the number of years of mining experience. For example, there was an inverse correlation between number of years mining and injury frequency (Spearman’s Rho 0.11, *p* < 0.0001). This was reflected in the injury rate, which was highest amongst miners with less than one year of experience (25.31 injuries per 100 person years), *versus* those with 1–<5 years (12.72 injuries per 100 person years) and 5–10 years of experience (5.10 injuries per 100 person years and those with over 10 years of experience (4.36 injuries per 100 person years). There was, however, no association between the age of miners and the occurrence of injury (*p* = 0.39). The plurality of the injuries (43.8%) occurred during the morning with 36.4% and 19.8% occurring in the afternoon and evening/night, respectively.

**Figure 3:**
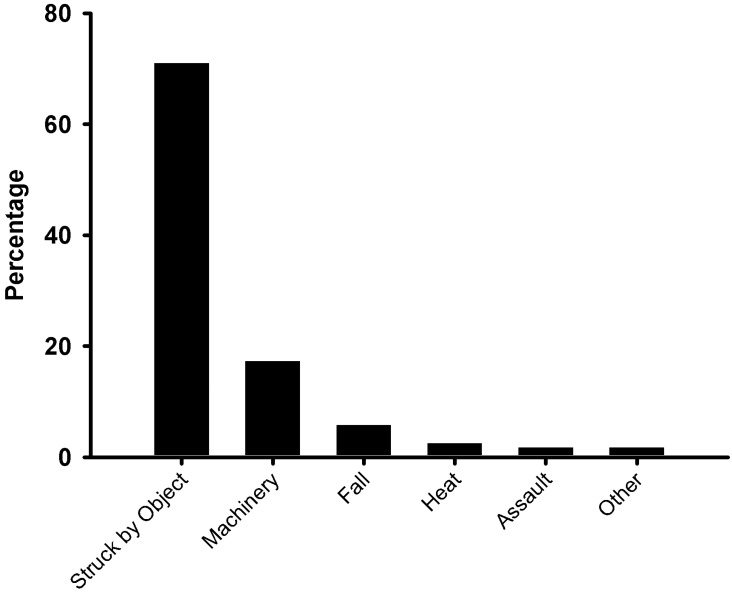
Self-reported causes of injury (from the 121 injury events) by the artisanal and small-scale gold miners from the Tarkwa (Ghana) study sites.

Over 70% of the injuries recorded were reported to be the direct result of miners being hit or struck by an object, followed by the use of machines or tools (17.3%), with other causes of less than four percent each (Figure 3). Of the 121 recorded injury events, the majority (57%) were lacerations, followed by puncture wounds (13%) and abrasions (11%) (Table 3). Nearly 70% of the injuries were to either the upper or lower limbs, with injuries to the head, eyes, ears or face contributing another 17%. Approximately 20% of all the injuries occurred to the feet.

In this study, the number of days lost from work was used as a measure of the severity of the injury sustained (International Labor Organization (ILO), 1998). Of the 121 recorded injury events, 30.6% of them were deemed mild (“no day lost” up to three days of absence from work), 34.7% were moderate (4–14 days of absence) and 34.7% were severe (absence for more than 14 days). Notably, 15% of injuries were severe enough to require a month or more of absence (Table 4).

### 3.4. Prevention and Training

Of the 95 workers who sustained injuries during the last 10 years, 25% believed that unusual work pressure played a role in their getting injured, 6% of them blamed their injury on distraction by personal issues, while the majority (69%) could not think of anything that could have directly contributed to their being injured.

Nearly two-fifths (36/95) of the injured believed their injuries could have been prevented. Of these 36 respondents, 18 (50%) believed this could have been achieved with the availability and proper use of personal protective equipment (PPE). For women, 60% believed the injury could have been prevented. The PPE mentioned by the 18 individuals (males and females) indicated above were safety boots (three), safety goggles (one) and gloves (two). Twelve (12) of them, however, did not specify the type of PPE they believed could have prevented the injury. A further seven (19.4%) were convinced that the injury could have been averted if they had been more careful in the discharge of their duties, while four (11%) thought having a safer work environment and reduced work pressures could have prevented those injuries from occurring. 

**Table 3:** Type and localization (body part) of injuries self-reported by artisanal and small-scale gold miners in the Tarkwa (Ghana) study sites according to age and duration of mining. The percentages are based on 121 self-reported injury events.

	Head	Eye/Ear/Face	Chest/Back	Upper Limb	Lower Limb	Other	Total
**Abrasions **	0.8	0.8	0.8	2.5	5	0.8	**10.7**
**Lacerations **	5.8	3.3	0.8	28.1	18.2	0.8	**57.0**
**Puncture wounds**	2.5	0.8	0.8	3.3	5	0.8	**13.2**
**Amputations**	0	0	0	2.5	0	0	**2.5**
**Dislocations **	0	0.8	0	0	3.3	0.8	**4.9**
**Fractures **	0	0	0.8	0.8	0	0.8	**2.4**
**Other **	0	2.5	2.5	0.8	0	3.3	**9.1**
**Total**	**9.1**	**8.2**	**5.7**	**38**	**31.5**	**7.3**	**100**

**Table 4:** Number of days lost following injury.

Number of days lost	Frequency (%)
0	19 (15.7)
1–3	18 (14.9)
4–7	21 (17.4)
8–14	21 (17.4)
15–30	24 (19.8)
>30	18 (14.9)

Thirty one percent of the 404 miners surveyed reported ever receiving any form of training on how to mine safely. Of the 95 miners who had been injured, 27 (28.4%) had received some form of training on safe mining practices. There was no association between reports of having received training and the occurrence of injuries (*p*-value = 0.54). Because most of those trained could not remember dates on which they received their trainings, it could not be determined whether training antedated their injuries or *vice versa*. Further, we were unable to determine the regularity and quality of training received.

### 3.5. Employer Attitudes 

The 33 participants who always or sometimes acted as employers were excluded from responding to questions about employers’ attitudes towards safety, thus leaving 371 respondents. About one quarter of the employees reported that their employers never seem to be interested in anything that has to do with the welfare or safety of their employees (Table 5). Forty-five (12.1%) of the miners had at one point in time or the other refused to work because of safety concerns. Some of the reasons cited for their refusal to work included the absence of personal protective equipment, faulty machines that remained unrepaired after several complaints and unsafe working environment due to slippery ground, weakened tunnel roofs and cracks in the floor of the pits.

**Table 5:** Responses by miners concerning the attitude of their employers towards safety issues in artisanal and small-scale gold mining communities in the Tarkwa (Ghana) region. Values in the table are percentages. For the final question, 9.4% indicated not having any complaints.

	Always	Sometimes	Never	No Answer
**Does your employer show concern about staff welfare?**	28.5	41	24.3	6.2
**Does your employer discuss safety issues with the staff?**	22.1	42.9	28.8	6.2
**Does your employer consider suggestions for improving workplace safety?**	19.9	40.7	28.3	11.1
**Are your concerns about safety being acted upon?**	21.1	36.7	26.1	6.7

## 4. Discussion

Small-scale gold mining is proliferating in many regions worldwide, yet there is little known about occupational hazards and injury factors within this sector. There are only a handful of studies, largely descriptive, and these vary greatly in methodology, making it difficult to generalize and make comparisons. Here, a cross-sectional study was conducted in the well-studied small-scale gold mining region of Tarkwa in Ghana. Our study revealed a number of important findings. Nearly one-quarter of the 404 miners interviewed reported getting injured over the previous 10 years, and the overall injury rate was calculated to be 5.39 per 100 person years. The rate was significantly higher for women (11.93 per 100 person years) and those with little mining experience (e.g., 25.31 per 100 person years for those with less than one year of work experience). The most injury-prone mining activities were excavation and crushing, and over 70% of the injuries were reported to be due to miners being hit by an object. The majority of the injuries were lacerations, and nearly 70% of the injuries were to the upper or lower limbs. More than two-thirds of the injuries resulted in miners missing more than four days of work. One-quarter of the injured workers believed that abnormal work pressure played a role in their injuries, and nearly two-fifths believed that their injuries could have been prevented, with many citing personal protective equipment as a solution. About one quarter of the employees reported that their employers never seemed to be interested in the welfare or safety of their employees, thus pointing to organizational weaknesses that may create unsafe working conditions. 

Few research studies have characterized injuries in small-scale gold mining communities, though the International Labour Organization [5] estimates that artisanal and small-scale mining operations worldwide experience six–seven-times more non-fatal accidents than large-scale operations. Given the informal nature of small-scale gold mining, formal reporting schemes do not exist, and this represents a major data gap. In the current study, the calculated injury overall rate of 5.39 per 100 person years is lower than the value (392 accidents per 100 person years) reported in a study of small-scale miners in Katanga, Democratic Republic of the Congo [19]. This latter study surveyed 180 miners and documented 392 accidents over the past one year, though unlike our study, it collected information on all injuries irrespective of whether it affected the miners’ ability to work or not. The latter study also contained information on fatalities, as well as permanent disabilities that made it impossible for those affected to continue mining. These methodological aspects likely account for much of the difference between the reported injury rates.

In the current study, the frequency of injury decreased with increasing number of working years, with only 6.3% of those who had worked for ≥20 years experiencing injuries. Furthermore, the injury rate was highest amongst miners with less than one year of experience (25.31 injuries per 100 person years). Thus, experience appears to play an important role in the prevention of injuries among the small-scale miners. Stojadinovic *et al*. [20] studied injuries among underground coal miners and also found the highest injury rates amongst miners with the least experience. These findings are in contrast to the work from Katanga (Democratic Republic of the Congo) in which artisanal miners with more than three years work experience reported more injuries than newer miners [19]. The setting and/or activities of the miners involved in these studies differ and may account for the discrepancies.

In the current study, the incurred injuries were severe enough to cause workers to miss workdays or significantly hamper their ability to carry out work tasks. Close to 16% of the injured workers did not have to miss work on account of the injury. Another 15% of injured workers lost between 1–3 days of work, while the vast majority (69%) lost more than three days of work as a result of the injury they suffered. The trend is quite similar to what was observed among artisanal miners in Katanga, where 50.8% of the injured lost three days or more from work [19]. 

As observed in other small-scale gold mining communities in Ghana [15], the workforce reported in the current study is predominantly young males with a median age of 32 years. However, a number of women are involved in the sector and face unique sensitivities, as shown previously in other Ghanaian mining communities [11,21]. Here, 32 women were interviewed, and their results are illuminating in terms of socio-demographics and work history. The women were slightly older than the men, but only 9.4% were single *versus* 25% of the men. The women were much less educated, with 56% reported having never attended school *versus* 6.5% of the men. Work duration for the females interviewed was not different from the males, but the type of activities varied. Whereas most of the men were involved with excavation and/or crushing, most of the women carried loads. The women interviewed reported 14 injury episodes over 117 person years, thus resulting in a calculated injury rate (11.93 injuries per 100 person years) that was significantly higher than that for the men. Gender-related disparities in small-scale gold mining communities require further attention.

In this study, most (71%) of the injuries resulted from miners being hit by objects in the course of work. This was followed by injuries resulting from the handling of mining tools/machinery (17%) and falls (5.8%). Moving or falling objects were reported to be the major cause of acute traumatic injuries among large-scale gold miners in Ghana [22]. Another Ghanaian study of workers within the formal mining sector reported falls as the major cause of accidents [23]. Amongst copper miners in Zambia, the handling of tools and materials (26%), falling of rocks and other objects (20%) and slips/falls (12%) were the major causes of injury [24]. Amongst artisanal miners in Katanga, D.R.C., the handling of tools (51.5%), handling of heavy loads (32.9%) and falls (11.5%) were the main causes of injury [19]. While there is no uniformity across these studies in terms of identifying a single major cause of injury, there are some consistently reported factors related to human error that warrant further investigation. It is worth noting that a survey of equipment-related injuries in the U.S. mining sector revealed that non-powered hand tools were most often associated with non-fatal injuries [25].

The upper and lower limbs were observed in the current study to be the most affected body parts, followed by injuries to the head. Most of the injuries were lacerations. Other studies on miners have found the upper and lower limbs to be the most affected [19,20]. In addition, the study by Elenge *et al*. [19] on small-scale miners documented that the most common types of injuries are bruises (50.2%), wounds (44.4%) and fractures (5.4%). 

In the current study, of those who were injured, nearly two-fifths believed their injuries could have been prevented, with 50% of those indicating that this could have been achieved with the availability, training and proper use of personal protective equipment. Over 80% of those who sustained injuries were without any sort of protective equipment at the time of their injury. This is a reflection of human error and the general low usage of PPEs among the small-scale miners surveyed that was observed anecdotally during the study. The number is similar to what was previously reported in a small-scale mining occupational exposure study from Ghana’s Upper East Region, in which 70% indicated that they never use personal protective equipment [15]. Nearly 20% of all injuries occurred to the feet, with about 9% affecting the head. These injuries to the head and feet might have been minimized if helmets and steel-toed boots were available and being used routinely by the miners. The role of education is unclear, but warrants more attention. Approximately 30% of the miners surveyed reported receiving some form of mine safety training, yet this does not seem to be uniformly available. However, there was no association between the occurrence of injuries and those who had received some form of training.

## 5. Conclusions 

Excluding fatalities and disabling injuries, this study observed a substantial injury rate of 5.39 per 100 person years. The injuries were mainly the result of falling objects and handling of tools/machinery and resulted in injuries mostly to the limbs and head. The activity of the miner and the number of years of mining (experience) were factors that significantly affected injury occurrence in this study. Excavation and crushing/grinding activities were found to be more likely to result in injuries compared to the other mining activities, while those who had been mining for a longer period of time were less likely to be injured compared to those who had been mining for shorter periods of time (less than three years). Our findings are almost certainly an underestimate of the true risk, because the cross-sectional study design utilized would miss injuries severe enough to force individuals to permanently retire from mining. This study was reliant on self-reported information. Moving ahead, it is necessary for such mining entities to keep formal records and information on employee safety and training. While the study was not specifically designed to evaluate interventions, our findings and observations suggest the need to emphasize methods that have been demonstrated to substantially reduce occupational injuries in many other settings. These include the greater use of PPE, such as helmets and steel-toed boots, to protect against injuries to the head and feet, more frequent and substantial training in health and safety and the consideration of apprenticeships to help their workers understand the hazards and how to avoid them through advice from more experienced workers. Further attention should focus on women who face disproportionate gender-related exposures. 
